# Bumetanide As a Candidate Treatment for Behavioral Problems in Tuberous Sclerosis Complex

**DOI:** 10.3389/fneur.2017.00469

**Published:** 2017-09-08

**Authors:** Chantal Vlaskamp, Simon-Shlomo Poil, Floor Jansen, Klaus Linkenkaer-Hansen, Sarah Durston, Bob Oranje, Hilgo Bruining

**Affiliations:** ^1^Department of Psychiatry, Brain Centre Rudolf Magnus, University Medical Centre Utrecht, Utrecht, Netherlands; ^2^CNCR, VU University Amsterdam, Amsterdam, Netherlands; ^3^Department of Child Neurology, Brain Centre Rudolf Magnus, University Medical Centre Utrecht, Utrecht, Netherlands

**Keywords:** tuberous sclerosis complex, tuberous sclerosis complex-associated neuropsychiatric disorders, hyperexcitability, bumetanide, electroencephalography, event-related potentials

## Abstract

**Background:**

Recent studies indicate excitatory GABA action in and around tubers in patients with tuberous sclerosis complex (TSC). This may contribute to recurrent seizures and behavioral problems that may be treated by agents that enhance GABAergic transmission by influencing chloride regulation.

**Case presentation:**

Here, we used the chloride transporter antagonist bumetanide to treat a female adolescent TSC patient with refractory seizures, sensory hyper-reactivity, and a variety of repetitive and compulsive behaviors.

**Methods:**

To evaluate the effect of bumetanide on behavior, auditory sensory processing, and hyperexcitability, we obtained questionnaire data, event-related potentials (ERP), and resting state EEG at baseline, after 3 and 6 months of treatment and after 1 month washout period.

**Discussion:**

Six months of treatment resulted in a marked improvement in all relevant behavioral domains, as was substantiated by the parent questionnaires. In addition, resting-state electroencephalography and ERP suggested a favorable effect of bumetanide on hyperexcitability and sensory processing. These findings encourage further studies of bumetanide on neuropsychiatric outcome in TSC.

## Introduction

This case report describes a 20-year-old female patient with tuberous sclerosis complex (TSC) characterized by a mild mental retardation, autism spectrum disorder (ASD), focal epileptic seizures, and a wide range of disabling behavioral problems. The behavioral problems became unmanageable and she presented to our outpatient clinic for treatment possibilities. The wide range of refractory behavioral problems and previous paradoxical response to valproic acid (VPA) led to the suspicion of depolarizing GABA activity.

## Background

Treatment in TSC is mostly aimed at controlling seizures, whereas there are few treatment options for the accompanying behavioral and neuropsychiatric problems at present. Irritability, sensory arousal, repetitive behaviors, sleeping problems, and deficits in social communication are very common in patients with TSC (1) and these so-called tuberous sclerosis-associated neuropsychiatric disorders (TAND) are often extremely debilitating, although rarely treated successfully. Pharmacotherapy to reduce TAND is mostly attempted with psychostimulants such as methylphenidate or antipsychotic treatments, both of which can have serious side-effects, such as lowering the seizure threshold (2). Treatment of TAND is presumably complicated by the variability and heterogeneity of TAND (3). However, syndrome specific mechanisms such as mTOR dysregulation may be a general target to reduce TAND across the TSC population.

Indeed, mTOR inhibitors are being tested as possible treatment for a wide variety of neurological sequelae of TSC. Although promising for seizure reduction, no clear effects on behavioral outcome have yet been established (4). Recent studies have suggested another avenue for treatment of neurological problems. It has been shown that chloride homeostasis may be disrupted in and around tuber tissue in TSC as indicated by altered expression levels of the chloride cotransporters NKCC1 and KCC2 (Na–K–2Cl cotransporter isoform 1 and K–Cl cotransporter isoform 2, respectively). These disturbances could be leading to (focal) depolarizing GABA activity and, consequently, altered balance between excitation and inhibition (E/I) in these regions (5, 6). These findings suggest that the NKCC1 chloride transporter antagonist bumetanide might enhance synaptic transmission by strengthening inhibition in TSC. Indeed, Talos et al. (6) showed that excitatory GABA activity in slice preparations of human tuber tissue was effectively attenuated by bumetanide *in vitro* (6).

Clinical benefit of bumetanide therapy has not yet been demonstrated in TSC but has been shown to reduce core behavioral problems in ASD (7, 8). The strong association of TSC with ASD further suggests that bumetanide might be able to improve behavioral problems in TSC. Additionally, we have previously suggested that (history of) a paradoxical reaction to GABAergic drugs may be a prognostic marker of depolarizing GABA activity, suggesting bumetanide may be efficacious (9). In the current study, bumetanide was administered to a 20-year-old female with TSC and a history of paradoxical reaction to VPA. Effects on behavior and sensory processing were assessed using questionnaires and a neurophysiological test battery.

## Case Presentation

A 20-year-old female patient with TSC presented to our clinic with a long history of neurodevelopmental problems characterized by moderate mental retardation (Total IQ ~50), ASD (pervasive developmental disorder not otherwise specified) according to the DSM-IV TR (diagnostic and statistical manual of mental disorders, fourth edition, text-revision) and focal epileptic seizures. The manifestation of TAND in this patient was characterized by intellectual disability, sensory hyper-reactivity, irritability, obsessive and compulsive behaviors, behavioral rigidity, and inflexibility, accompanied by sleeping problems.

Family history showed second-degree history of ASD. Birth was complicated by meconium aspiration that resulted in several days of assisted ventilation. The first established developmental problems were motor- and speech delay at toddler age. TSC was suspected because of focal seizures from the age of 9 months, skin pigment stains, and structural anomalies on brain imaging. Genetic investigation confirmed a spontaneous deleterious mutation in the TSC1 gene. Subsequent MRI scans showed subcortical tubers and subependymal noduli in both hemispheres. Since childhood, the patient experienced focal to bilateral tonic-clonic seizures 1–2 times a year and focal seizures with behavioral arrest and automatisms 1–5 times daily. Localization of interictal epileptiform discharges was left frontally. Previous treatment with various antiepileptic drugs (AED) did not sufficiently control seizures. The patient had experienced a paradoxical response (evident increase instead of decrease of irritability and anxiety) to VPA. AED regime at time of the study consisted of oxcarbazepine monotherapy.

The previous paradoxical response to VPA and daily burden of behavioral problems and seizures led us to suspect depolarizing GABA activity. Therefore, we treated the patient with 0.5 mg bumetanide twice daily [according to Lemonnier et al. (8)] for a 6-month trial followed by a 1-month washout period to substantiate efficacy. Parents of the patient gave consent for off-label treatment. Monitoring of treatment effect included an extensive evaluation of behavioral questionnaires and EEG/event-related potentials (ERP) measurements. Blood tests and physical examinations were carried out as described previously (9). Transient hypokalemia was successfully treated with potassium supplements. Treatment did not cause other disturbances or discomfort due to diuretic effects. The patient received no concurrent therapies or interventions during the course of treatment, other than continuation of oxcarbazepine (a sodium channel inhibitor).

## Methods

### Questionnaires

Clinical Global improvement (CGI) was assessed by the treating psychiatrist. Questionnaires included the Social Responsiveness Scale (SRS) (10), the Sensory Profile (SP-NL) (11), the Repetitive behavior scale-Revised (RBS-R) (12), the Aberrant behavior checklist (ABC) (13), and the Behavior rating Inventory of executive function (BRIEF) (14) and were filled in by both parents and tutor.

### ERP Measurements

Auditory sensory processing was assessed using a P50 suppression task to measure sensory gating and a passive oddball paradigm to measure mismatch negativity (MMN). Both tasks originate from the Copenhagen Psychophysiological test battery (15, 16), which additionally consisted of a startle paradigm (PPI) and a selective attention task. All testing was performed with a 64 electrode BioSemi set-up according to the 10–20 system. Sampling started as soon as the paradigms were started and lasted to the end of it (continuous recording). The sample frequency was 2,048 Hz, and a low-pass setting of 1/5 of the AD rate. All stimuli were presented binaurally through tubal insert ear phones (EARtone^®^, Etymotic Research), by a computer running Presentation^®^ software (Neurobehavioral Systems Inc.).

#### P50 Paradigm

The P50 paradigm consisted of three experimental blocks, each consisting of 40 pairs of identical bursts of (1.5 ms and 80 dB) white noise, with an instantaneous rise time, an inter-stimulus interval (ISI) of 500 ms, and a fixed inter-trial interval of 10 s. The subject was asked to count the stimuli she was presented and to stay awake. The total duration of the P50 task was approximately 21 min.

#### MMN Paradigm

The MMN paradigm consisted of 1,800 stimuli. The paradigm consisted of four types of stimuli: in 82% of the cases, a standard tone with a frequency of 1,000 Hz, intensity of 75 dB, and duration of 50 ms was presented. Within this sequence of standard stimuli, three types of deviants were presented, each with a probability of 6% and intensity of 75 dB: frequency deviants of 1,200 Hz and 50 ms, duration deviants of 1,000 Hz and 100 ms, and frequency-duration deviants of 1,200 Hz and 100 ms. The ISI was randomized between 300 and 500 ms. The subject was asked to ignore the stimuli while watching a silent movie. The total duration of the MMN task was approximately 15 min.

#### ERP Analysis

Analysis and processing of the EEG signal was carried out using Brain Electrical Source Analysis (BESA) software (version 5.2.4, MEGIS Software GmbH, Gräfelfing, Germany). Only data from relevant electrodes were processed and analyzed; i.e., Cz for P50 and FCz for MMN. Processing of the data started with re-sampling from the original 2,048 to 250 Hz to allow easier file handling. Second, the data were corrected for eye-artifacts by using the adaptive method of BESA. Third, the data were epoched (from 100 ms prestimulus to 400 ms post-stimulus for P50, and 900 ms post-stimulus for MMN), and corrected for movement (or other paradigm unrelated) artifacts by removing epochs that contained amplitude differences between maximum and minimum exceeding 75 µV in the relevant scoring windows. Subsequently, the data were band-pass filtered (high-pass: 1 Hz, low-pass: 70 Hz for P50, 40 Hz for MMN). For MMN, each of the three deviant types was expressed as the average ERP to the relevant deviant stimuli, subtracted with the average ERP to standard stimuli for each subject separately. Linked mastoids were used as a reference. Finally, MMN amplitudes were scored as the minimum amplitude within a window between 100 and 230 ms.

For P50 analysis, we used an average reference. P50 amplitude was defined as the largest trough to peak amplitude within an interval of 40–90 ms following the first (conditioning or “C”) stimulus in each paired click. The P50 amplitude following the second (testing or “T”) stimulus was identified as the largest trough to peak amplitude within an interval of 10 ms of the latency of the maximum P50 amplitude to the C-stimulus. P50 suppression was expressed as the ratio “T/C.”

### Resting-State EEG Measurements

Resting-state EEG was measured during 3 min eyes-closed rest using BioSemi with a sampling frequency of 2,048 Hz (resampled offline to 512 Hz for analysis) with no hardware filters. Analysis was performed using the Neurophysiological Biomarker Toolbox (17). The signals were reviewed and transient artifacts were cut out. As a measure of long-range temporal correlations, we applied detrended fluctuation analysis (DFA) [see Linkenkaer-Hansen et al. (18)] to the amplitude envelop of band-pass filtered signal (FIR filter with a Blackman window, transition bandwidth 1 Hz) in the bands Alpha 8–13 and 6–13 Hz. A DFA exponent of 0.5 implies that oscillations amplitudes fluctuate in an uncorrelated fashion over time. When exponents increase from 0.5 toward 1.0, they signify increasing strength of long-range temporal correlations. Power spectral density was calculated using Welch’s method with 8 s hamming windows. Relative power was calculated as the bandwidth power normalized by the power in the 1–45 Hz band.

## Results

No significant change in seizure frequency or severity was observed. From approximately 1 month of treatment, marked improvements in social responsiveness, attention, reduced irritability, and restlessness were noted by both parents. In addition, sleep efficiency was improved. Parental observations were substantiated through questionnaires showing marked improvement in behavior following bumetanide treatment.

**Figure 1:**
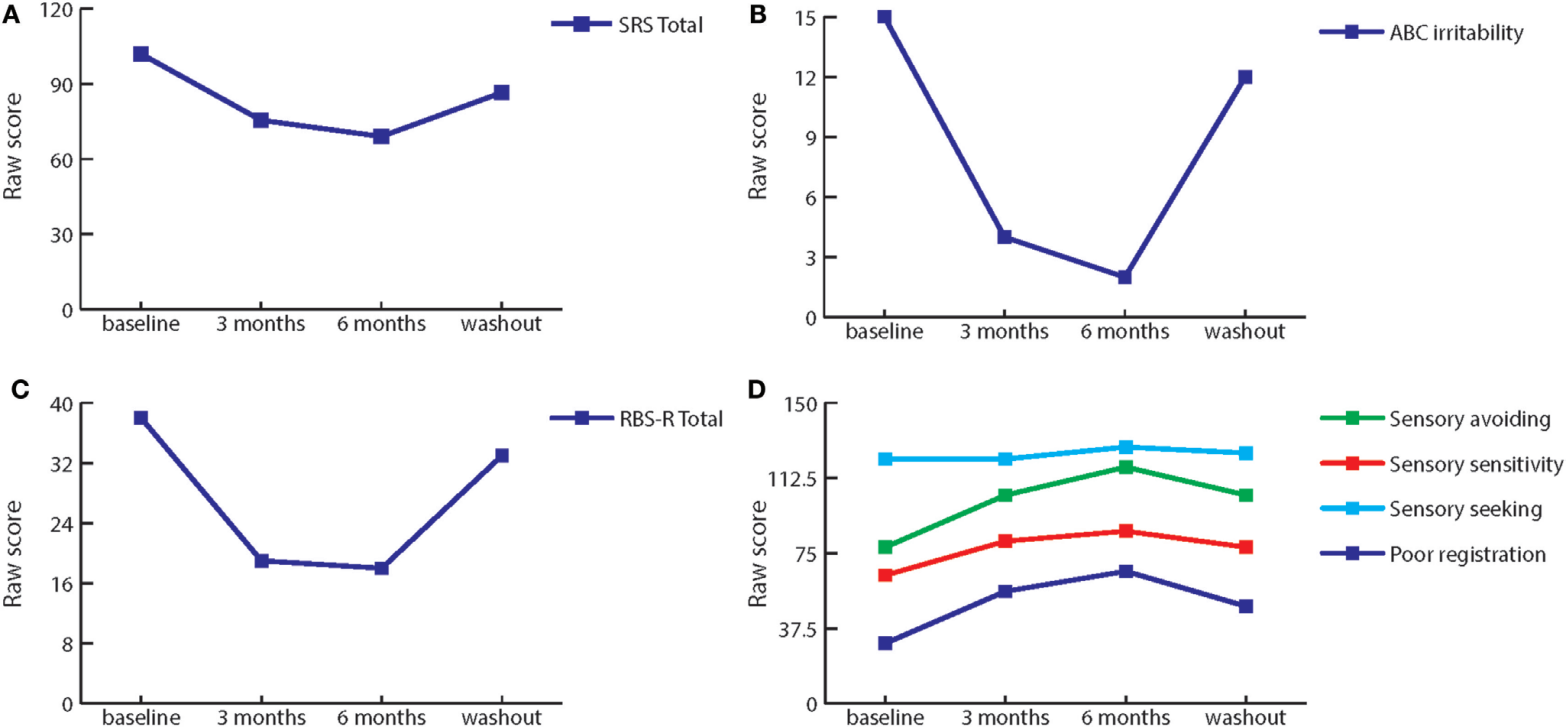
Effect of bumetanide on behavior. **(A)** Total score of the social responsiveness scale is decreased by treatment with bumetanide, the effect is removed at washout. **(B)** Irritability, as measured by the Abberant behavior checklist subscale, is markedly decreased by bumetanide treatment. At washout, irritability increases again. **(C)** Repetitive behavior is decreased by bumetanide treatment, as indicated by total score on the repetitive behavior scale-revised (RBS-R). Washout again removes this effect. **(D)** Similarly, sensory behavior as measured by Sensory Profile subscales improves during 3 and 6 months of bumetanide. Again, washout reverses the effect.

The greatest improvements (>50% from baseline) after 3 and 6 months of treatment were seen on (subscales of) the Sensory Profile (SP-NL), ABC, SRS, and RBS-R. In addition, improvements in behavior were also seen on the Behavior Rating Inventory Executive Function questionnaire (BRIEF) rated by the tutor of the patient. Figure 1 illustrates the most pronounced behavioral improvements, Table 1 shows the measured scores on all behavioral questionnaires at baseline, during treatment and at washout, and percentage of improvement after treatment.

**Table 1:** Questionnaire scores (raw scores).

Questionnaires—raw scores	Baseline	3 months	6 months	Washout	Improvement 3 months (%)	Improvement 6 months (%)
CGI	6	4	3	3	33.33	50.00
SP-NL poor registration^a^	30	56	66	48.5	86.66	120.00
SP-NL Sensory seeking^a^	122	122	128	125	0	2.46
SP-NL Sensory sensitivity^a^	64	81	86	78	26.56	21.88
SP-NL Sensory avoiding^a^	78	104	118	104	33.33	51.28
SRS total	129	79	66	100	38.76	48.84
SRS social awareness	17	10.5	9	10.5	38.24	47.06
SRS social cognition	29	17	14	22.5	41.38	51.72
SRS social communication	48	29	22	36	39.58	54.17
SRS social motivation	11	8	7	12	27.27	36.36
SRS restricted interests	24	14.5	14	19	39.58	41.67
ABC total	71	17.5	16	45	75.35	77.46
ABC irritability	15	4	2	12	73.33	86.67
ABC lethargy	19	6	4	12.5	68.42	78.95
ABC stereotypy	6	1.5	2	1.5	75.00	66.67
ABC hyperactivity	21	4	5	13	80.95	76.19
ABC inappropriate speech	10	2	3	6	80.00	70.00
RBS-R total	36	19	18	33	47.22	50.00
RBS-R stereotyped behavior	0	0	0	0.5	0.00	0.00
RBS-R self-injurious behavior	1	1	3	2	0.00	−200.00
RBS-R compulsive behavior	10	3.5	2	5	65.00	80.00
RBS-R routine behavior	9	3.5	3	9	61.11	66.67
RBS-R sameness behavior	13	8.5	7	12.5	34.62	46.15
BRIEF total parents (P)	178	143.5	129	149	19.38	27.53
BRIEF inhibition P	26	22	19	21.5	15.38	26.92
BRIEF shift P	22	16.5	16	18	25.00	27.27
BRIEF emotional control P	27	18.5	17	21	31.48	37.04
BRIEF initiate P	16	15.5	13	15.5	3.13	18.75
BRIEF working memory P	25	19	18	20.5	24.00	28.00
BRIEF plan/organize P	25	20	17	20	20.00	32.00
BRIEF organization materials P	16	13	11	14	18.75	31.25
BRIEF monitor P	21	19	18	18.5	9.52	14.29
BRIEF total school (S)	191	163	151	179	14.66	20.94
BRIEF inhibition S	30	26	24	28	13.33	20.00
BRIEF shift S	29	22	20	25	24.14	31.03
BRIEF emotional control S	24	21	17	24	12.50	29.17
BRIEF initiate S	18	17	15	14	5.56	16.67
BRIEF working memory S	26	22	22	25	15.38	15.38
BRIEF plan/organize S	20	15	16	20	25.00	20.00
BRIEF organization materials S	19	15	16	17	21.05	15.79
BRIEF monitor S	25	25	21	26	0.00	16.00

*Colored rows indicate greatest improvement after 3–6 months (i.e., 50%+)*.

*^a^Sensory profile subscales: higher scores are more toward normality (except for sensory seeking)*.

*CGI, clinical global improvement; SP-NL, sensory profile, Dutch edition; SRS, social responsiveness scale; ABC, aberrant behavior checklist; RBS-R, repetitive behavior scale-revised; BRIEF, behavior rating inventory executive function*.

**Figure 2:**
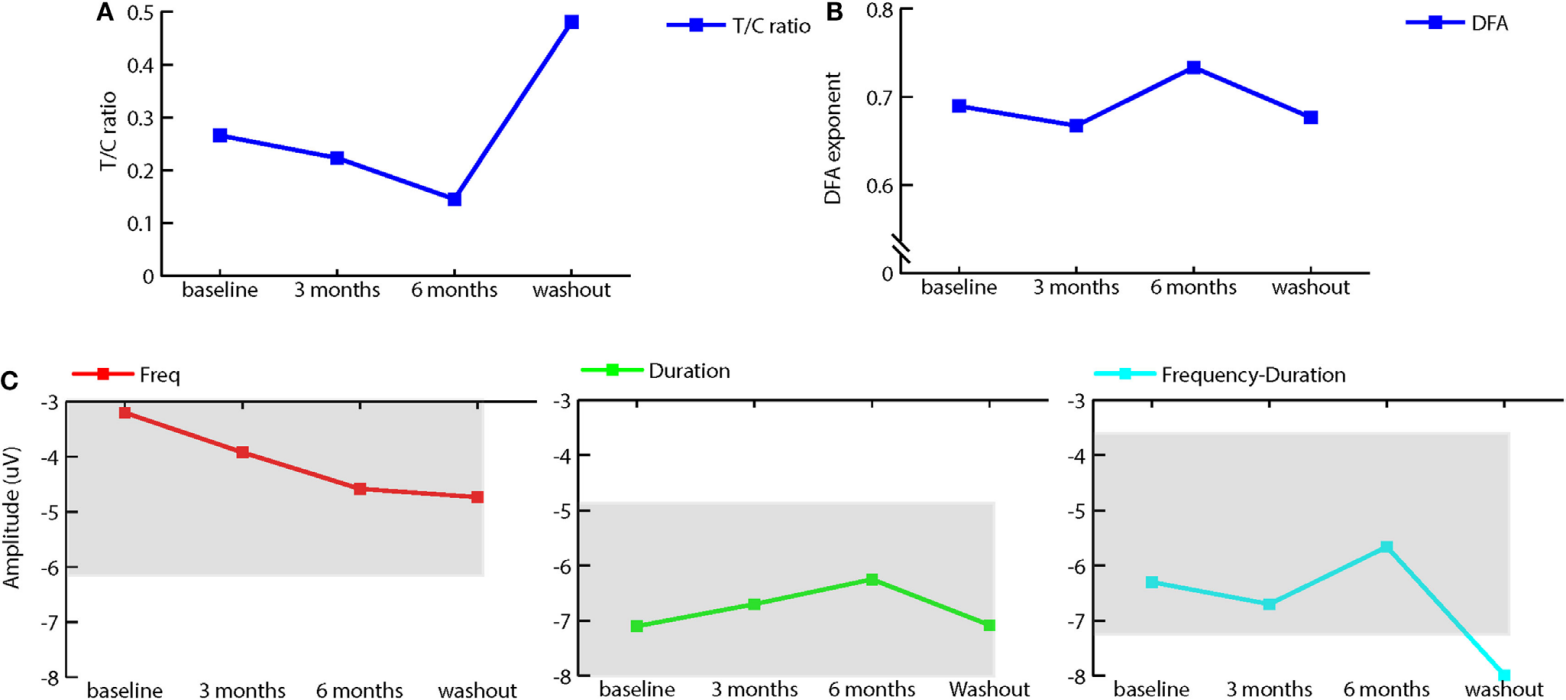
Effect of bumetanide on event-related potentials and resting-state EEG. **(A)** T/C ratio illustrating sensory gating (as measured by the P50 suppression paradigm) is increased (lower T/C) after 3 and 6 months of bumetanide treatment. Washout decreases sensory gating again. **(B)** Average detrended fluctuation analysis (DFA) exponents at different measurement time points (median across all channels) in the extended 6–13 Hz long-range temporal correlations (DFA exponents) are strengthened after 6 months bumetanide treatment. **(C)** Mismatch negativity for frequency (C1), duration (C2), and frequency-duration (C3) deviant. Frequency MMN amplitude increases after 6 months treatment, duration, and frequency-duration MMN amplitude show a decrease after 6-month treatment. Washout removes this effect. Gray areas represent normal range, ±1 SD from healthy population as in Rydkjaer et al. (19). Note: for duration MMN, normal range goes up to −11.98 μV.

To investigate physiological effects of treatment, we measured ERP using standard EEG paradigms of P50 suppression to assess sensory gating and a passive auditory oddball paradigm to measure mismatch negativity (MMN). In addition, we recorded resting-state EEG before and after treatment and washout. Sensory gating increased with bumetanide, followed by a decrease after washout (see Figure 2A). MMN amplitude to frequency deviants slightly increased after treatment, whereas the amplitude of duration and frequency-duration MMN slightly decreased. Again, washout appeared to reverse these effects (see Figure 2C). It should be noted that sensory gating as well as MMN were within normal ranges at baseline and during treatment (19).

Power spectrum analysis of resting-state EEG showed that the peak frequency of alpha activity was around 7 Hz, which is lower than in the normal population (8–13 Hz). No pronounced effects of bumetanide on peak or alpha power were observed, although (extended) alpha power was highest after 6 months of treatment and decreased again at washout. DFA measures the strength of long-range temporal correlations (17) and has previously been coupled to changes in the excitatory/inhibitory balance (20, 21). We applied this analysis as a possible proxy of changes in overall E/I imbalances of GABAergic enhancement through bumetanide. DFA in extended alpha band (6–13 Hz) showed an increase of 0.04 (median across channels) in the DFA exponent after 6 months of bumetanide treatment and a decrease at washout (see Figure 2B). This increase in the DFA exponent is on the order of magnitude of the effects seen in other brain disorders, such as Alzheimer’s disease (22) and schizophrenia (23).

## Discussion

This case describes beneficial effects of treatment with bumetanide on behavior in a 20-year-old female diagnosed with TSC. This provides clinical support of preclinical studies that suggested that neuronal disinhibition (immature GABA activity) may be a mechanism contributing to TSC pathogenesis. Various domains of behavioral functioning improved during 6 months of bumetanide treatment, as indicated by both parents and scores on behavioral questionnaires rated by parents as well as the tutor of the patient.

Although this is merely a case report, some of the neurophysiological measurements appear to support a positive effect of bumetanide treatment on neuronal inhibition. Most notably, the strength of long-range temporal correlations (DFA measurement) was normalized during treatment, an effect that disappeared during the washout period. This finding is in line with suggestions of an effect of bumetanide on the excitatory/inhibitory balance, as modeling and clinical studies have shown less strong long-range temporal correlations when the E/I balance is impaired (17, 20, 22). Sensory gating and MMN also demonstrated a possible positive effect of bumetanide, although this should be interpreted with caution due to lack of experience with individual ERP assessment.

Washout measurements suggest that bumetanide did not result in continued therapeutic effects when administration ceased in this patient, as behavior and DFA (and possibly ERPs) deteriorated after discontinuation of bumetanide.

Our group previously reported effectiveness of bumetanide in a child with ASD and epilepsy with a previous paradoxical reaction to GABAergic drugs. This girl also suffered from a structural brain lesion in the temporal lobe (9). Indeed, temporal lobe epilepsy has been linked to elevated expression of the chloride importer NKCC1, the specific target of bumetanide (24). In addition, increased NKCC1 expression has been found in cortical dysplasia specimens from humans with focal epilepsy related to those found in TSC (6). The current study, therefore, provides further preliminary evidence that neuronal disinhibition constitutes a treatment target in to cortical dysgenesis-related neurodevelopmental disorders, especially when paradoxical responses to GABAergic drugs have been noted.

## Concluding Remarks

This case report confirms that bumetanide may be effective in reducing the high burden of behavioral problems in TSC, at least for some patients. More extensive trials are required to confirm the efficacy of bumetanide in TSC.

## Ethics Statement

The patient and her parents provided verbal and written consent for publication of this case report.

## Author Contributions

CV: conception, data acquisition/collection, data analyses and interpretation of the work, drafting, and revision of the work. SS: data analyses, interpretation and revision of the work. FJ: patient supervision, interpretation, and revision of the work. KL-H, SD, BO: interpretation and revision of the work. HB: patient supervision, drafting, interpretation, and revision of the work.

## Conflict of Interest Statement

The authors declare that the research was conducted in the absence of any commercial or financial relationships that could be construed as a potential conflict of interest.

## References

[1] Curatolo P Moavero R de Vries PJ. Neurological and neuropsychiatric aspects of tuberous sclerosis complex. Lancet Neurol (2015) 14(7):733–45. 10.1016/S1474-4422(15)00069-1 26067126

[2] Asato MR Hardan AY. Neuropsychiatric problems in tuberous sclerosis complex. J Child Neurol (2004) 19(4):241–9. 10.1177/088307380401900401 15163088

[3] de Vries PJ Whittemore VH Leclezio L Byars AW Dunn D Ess KC Tuberous sclerosis associated neuropsychiatric disorders (TAND) and the TAND checklist. Pediatr Neurol (2015) 52(1):25–35. 10.1016/j.pediatrneurol.2014.10.004 25532776 PMC4427347

[4] de Vries PJ. Targeted treatments for cognitive and neurodevelopmental disorders in tuberous sclerosis complex. Neurotherapeutics (2010) 7(3):275–82. 10.1016/j.nurt.2010.05.001 20643380 PMC5084231

[5] Ruffolo G Iyer A Cifelli P Roseti C Muhlebner A van Scheppingen J Functional aspects of early brain development are preserved in tuberous sclerosis complex (TSC) epileptogenic lesions. Neurobiol Dis (2016) 95:93–101. 10.1016/j.nbd.2016.07.014 27425893

[6] Talos DM Sun H Kosaras B Joseph A Folkerth RD Poduri A Altered inhibition in tuberous sclerosis and type IIb cortical dysplasia. Ann Neurol (2012) 71(4):539–51. 10.1002/ana.22696 22447678 PMC3334406

[7] Grandgeorge M Lemonnier E Degrez C Jallot N. The effect of bumetanide treatment on the sensory behaviours of a young girl with Asperger syndrome. BMJ Case Rep (2014) 2014:bcr2013202092. 10.1136/bcr-2013-202092 24488662 PMC3912375

[8] Lemonnier E Degrez C Phelep M Tyzio R Josse F Grandgeorge M A randomised controlled trial of bumetanide in the treatment of autism in children. Transl Psychiatry (2012) 2:e202. 10.1038/tp.2012.124 23233021 PMC3565189

[9] Bruining H Passtoors L Goriounova N Jansen F Hakvoort B de Jonge M Paradoxical benzodiazepine response: a rationale for bumetanide in neurodevelopmental disorders? Pediatrics (2015) 136(2):e539–43. 10.1542/peds.2014-4133 26216321

[10] Constantino JN Davis SA Todd RD Schindler MK Gross MM Brophy SL Validation of a brief quantitative measure of autistic traits: comparison of the social responsiveness scale with the autism diagnostic interview-revised. J Autism Dev Disord (2003) 33(4):427–33. 10.1023/A:1025014929212 12959421

[11] Tomchek SD Dunn W. Sensory processing in children with and without autism: a comparative study using the short sensory profile. Am J Occup Ther (2007) 61(2):190–200. 10.5014/ajot.61.2.190 17436841

[12] Lam KS Aman MG. The repetitive behavior scale-revised: independent validation in individuals with autism spectrum disorders. J Autism Dev Disord (2007) 37(5):855–66. 10.1007/s10803-006-0213-z 17048092

[13] Rojahn J Aman MG Matson JL Mayville E The aberrant behavior checklist and the behavior problems inventory: convergent and divergent validity. Res Dev Disabil (2003) 24(5):391–404. 10.1016/S0891-4222(03)00055-6 12951135

[14] Huizinga M Smidts DP. Age-related changes in executive function: a normative study with the Dutch version of the Behavior Rating Inventory of Executive Function (BRIEF). Child Neuropsychol (2011) 17(1):51–66. 10.1080/09297049.2010.509715 21218296

[15] Oranje B Rasmussen H Ebdrup BH Glenthoj BY Selective attention and mismatch negativity in antipsychotic-naïve, first-episode schizophrenia patients before and after six months of antipsychotic monotherapy. Psychol Med (2017) 47:2155–65. 10.1017/S0033291717000599 28443529

[16] Madsen GF Bilenberg N Jepsen JR Glenthoj B Cantio C Oranje B. Normal P50 gating in children with autism, yet attenuated P50 amplitude in the Asperger subcategory. Autism Res (2015) 8(4):371–8. 10.1002/aur.1452 25599888

[17] Hardstone R Poil SS Schiavone G Jansen R Nikulin VV Mansvelder HD Detrended fluctuation analysis: a scale-free view on neuronal oscillations. Front Physiol (2012) 3:450. 10.3389/fphys.2012.00450 23226132 PMC3510427

[18] Linkenkaer-Hansen K Nikouline VV Palva JM Ilmoniemi RJ. Long-range temporal correlations and scaling behavior in human brain oscillations. J Neurosci (2001) 21(4):1370–7. 11160408 10.1523/JNEUROSCI.21-04-01370.2001 PMC6762238

[19] Rydkjaer J Mollegaard Jepsen JR Pagsberg AK Fagerlund B Glenthoj BY Oranje B. Mismatch negativity and P3a amplitude in young adolescents with first-episode psychosis: a comparison with ADHD. Psychol Med (2017) 47(2):377–88. 10.1017/S0033291716002518 27776572

[20] Poil SS Hardstone R Mansvelder HD Linkenkaer-Hansen K. Critical-state dynamics of avalanches and oscillations jointly emerge from balanced excitation/inhibition in neuronal networks. J Neurosci (2012) 32(29):9817–23. 10.1523/JNEUROSCI.5990-11.2012 22815496 PMC3553543

[21] Poil SS Jansen R van Aerde K Timmerman J Brussaard AB Mansvelder HD Fast network oscillations in vitro exhibit a slow decay of temporal auto-correlations. Eur J Neurosci (2011) 34(3):394–403. 10.1111/j.1460-9568.2011.07748.x 21692883

[22] Montez T Poil SS Jones BF Manshanden I Verbunt JP van Dijk BW Altered temporal correlations in parietal alpha and prefrontal theta oscillations in early-stage Alzheimer disease. Proc Natl Acad Sci U S A (2009) 106(5):1614–9. 10.1073/pnas.0811699106 19164579 PMC2635782

[23] Nikulin VV Jonsson EG Brismar T. Attenuation of long-range temporal correlations in the amplitude dynamics of alpha and beta neuronal oscillations in patients with schizophrenia. Neuroimage (2012) 61(1):162–9. 10.1016/j.neuroimage.2012.03.008 22430497

[24] Huberfeld G Wittner L Clemenceau S Baulac M Kaila K Miles R Perturbed chloride homeostasis and GABAergic signaling in human temporal lobe epilepsy. J Neurosci (2007) 27(37):9866–73. 10.1523/JNEUROSCI.2761-07.2007 17855601 PMC6672644

